# Scale-down bioreactors—comparative analysis of configurations

**DOI:** 10.1007/s00449-025-03182-w

**Published:** 2025-06-05

**Authors:** Prasika Arulrajah, Anni Elina Lievonen, Dilara Subaşı, Subhashree Pagal, Dirk Weuster-Botz, Anna-Lena Heins

**Affiliations:** 1 Chair of Biochemical Engineering, School of Engineering and Design, Technical University of Munich, Boltzmannstr. 15, 85748 Garching, Germany; 2 Institute of Bioprocess and Biosystems Engineering, Hamburg University of Technology, Denickestr. 15, 21073 Hamburg, Germany; 3 TUM Campus Straubing for Biotechnology and Sustainability, Technical University of Munich, Petersgasse 5, 94315 Straubing, Germany

**Keywords:** Bioreactor, Gradients, Scale-down, Fermentation, Bioprocess, *E. coli*, *C. glutamicum*, *S. cerevisiae*, CHO

## Abstract

In large-scale bioprocesses, gradients in pH, dissolved oxygen level (DO), and substrate concentrations can decrease bioprocess efficiency. Scale-down bioreactors, be it single stirred-tank bioreactors with a special feeding regime, multi-compartment bioreactors, or combinations of bioreactors, offer a promising lab-scale solution for comprehending these gradients, as they allow adjustment of gradients without incurring high costs. However, critical challenges arise when transitioning from large-scale to scale-down bioreactors. Chief among these is realistically approaching the gradient conditions of large-scale bioreactors and choosing appropriate scale-down bioreactor configurations. This review paper begins by addressing the gradients encountered in large-scale bioreactors. Afterward, various types of scale-down bioreactors are characterized and compared, highlighting their advantages and disadvantages. The suitability of scale-down bioreactors is analyzed by examples of bioprocesses with different microorganisms and mammalian cells to underscore the complexities inherent in scale-down bioprocesses and emphasize the influence of cellular responses to these conditions. Finally, the potential of miniaturized and microfluidic bioreactors is briefly discussed for future application in scale-down studies.

## Introduction

It is imperative to transition from the current fossil-based industries that significantly contribute to climate change to industries based on renewable resources and a circular (bio)economy. To facilitate the shift, it is important to successfully operate large-scale bioprocesses obtaining high volumes and products with low selling prices [42]. Implementing such bioprocesses in prominent bulk sectors such as chemical industries is essential [67] and tremendous research has been performed to broaden the scope of bio-based products and to demonstrate proof of principle for new bioproducts [97].

However, realizing these as large-scale bioprocesses presents challenges so that to date, only a modest number have been successfully scaled up [79]. Early stages of bioprocess development are mostly conducted in laboratory-scale stirred-tank bioreactors (STRs), typically ranging from 0.5 to 10 L in volume [41]. The laboratory-scale volumes are, in the best case, scaled-up to industrial-scale volumes of several 100 m^3^ following the economy-of-scale principle [14]. During scale-up, numerous physical, chemical, and biologic factors might substantially disturb the bioprocess performance [87]. One notable challenge is gradients of, e.g., substrate concentration, dissolved oxygen level, or carbon dioxide often observed in large-scale bioreactors, which leads to microbial and environmental inhomogeneities [18, 42].

In laboratory-scale bioreactors, such as STRs, which are most commonly used in industrial production processes in addition to bubble column bioreactors, mixing is highly efficient. In smaller systems, mixing times are typically very short (< 5 s) [32], preventing the formation of gradients. However, as the volume increases, the mixing sufficiency decreases and gradients of process parameters are likely formed in the bioreactor, since the mixing time in large-scale can range from tens to 100 s of seconds [15, 93]. Thus, the mixing time can be notably longer than relevant cellular reaction time, which can be in the magnitude of seconds on transcriptome level [63]. In practice, the presence of gradients create multiple distinct microenvironments that cells must navigate and adapt to as they move through the bioreactor [30].

Bioprocess gradients can induce various responses of the cells leading to phenotypic population heterogeneity, which often results in decreased key performance indicators (KPIs) such as reduced productivity of the process, increased byproduct concentrations, and decreased biomass yield [21, 30]. Phenotypic population heterogeneity is a phenomenon when single cells within a population respond differently to environmental fluctuations, regardless of originating from isogenic cultures. These individual adaptation processes make the cell population potentially more robust and allow cells to cope with fluctuating environments [30]. For instance, it can lead to the selection of cells that exhibit greater resilience to stress, resulting in an overall enhancement of population fitness and improving the robustness of the bioprocess [17, 21, 51].

KPIs also worsen due to environmental heterogeneities (e.g., substrate, oxygen), for example, 20% reduction in biomass yield (Y_X/S_) of the β-galactosidase production process with *Escherichia coli* grown on glucose was found due to scaling-up the bioprocess from 3 to 9000 L [16]. To illustrate the problem further, in a fed-batch bioprocess with baker’s yeast grown on molasses, the final biomass concentration increased by 7% when the large-sale process was scaled-down from 120 m^3^ to 10 L with an identical strain, medium, and process control [26].

Scale-down studies can be conducted to investigate the effect of gradients exhibited in large-scale bioreactors caused by inadequate mixing on cellular physiology. In such studies, the gradients of the large-scale bioprocesses are mimicked in laboratory-scale bioreactors on a liter-scale, sometimes complemented with simulations (e.g., [12, 19]). Applying scale-down bioreactors effectively decreases the development time by allowing a better understanding of large-scale gradients and their effect on bioprocess performance as well as by enabling a notably higher precision and reliability during bioprocess scale-up [67].

This review paper begins by addressing the gradients encountered in large-scale bioreactors. Subsequently, various types of scale-down bioreactor configurations are characterized, and their advantages and disadvantages are discussed. The suitability of these configurations is then evaluated through selected cases, emphasizing the influence of cellular responses to fluctuating environmental conditions. Finally, the review highlights the potential of miniaturized and microfluidic bioreactors as emerging tools for future scale-down applications.

## Gradients in large-scale bioreactors

**Fig. 1:**
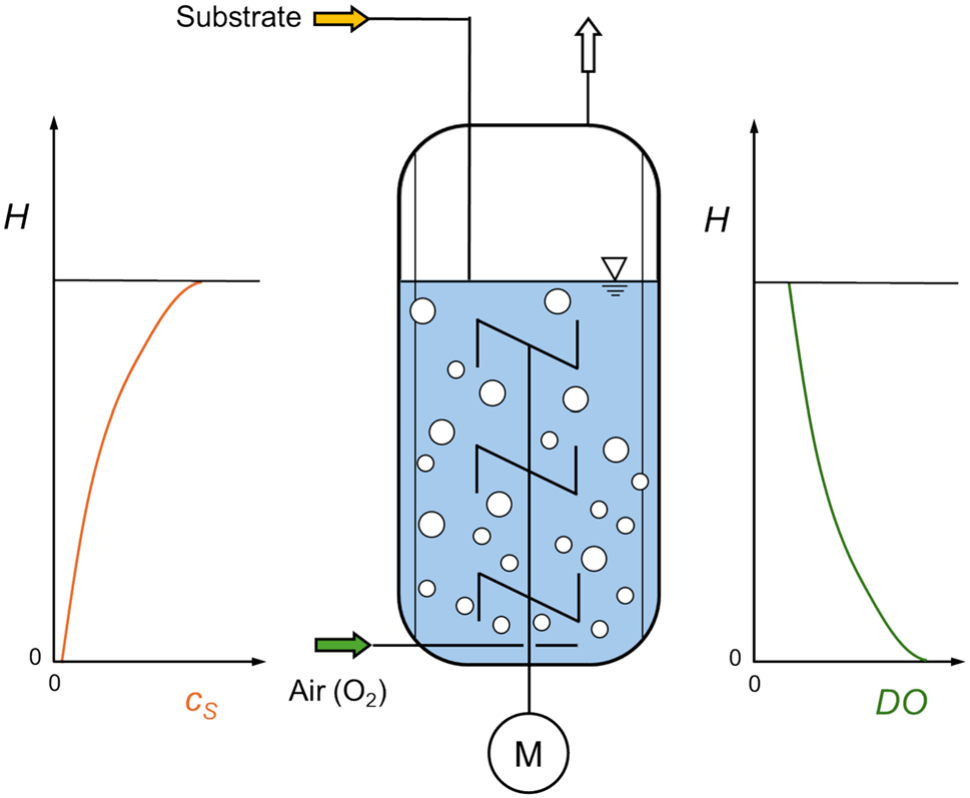
Principal scheme of the substrate (left) and dissolved oxygen (DO) concentration gradients (right) as a function of the liquid height *H* in large-scale stirred-tank bioreactors with the feeding of the concentrated substrate at the top (aerobic bioprocess)

Gradients in large-scale bioreactors occur in a wide range of bioprocesses such as anaerobic fermentations, photo-bioreactors, and aerobic fermentations [45, 46, 71, 82]. The most common ones in large-scale bioreactors are substrate and dissolved oxygen gradients (DO, Fig. 1), consequently making them the most studied [22]. Furthermore, gradients of pH, temperature, and dissolved carbon dioxide (CO_2_/HCO_3_^−^) are present and have been studied [14, 18]. These inhomogeneities arise because larger bioreactors exhibit longer mixing times due to reduced mixing efficiency, creating transient zones where cells encounter fluctuating conditions as they move though, e.g., oxygen-depleted or substrate-rich regions. Those gradients can alter microbial metabolism and reduce product yields due to exposure to different microenvironments [94].

Computational Fluid Dynamics (CFD) simulations have become indispensable, providing insights into the complex fluid dynamics and mass transfer phenomena that drive gradient formation. However, one significant challenge remains the accurate description of the biologic reactions to dynamically changing environmental conditions, which cannot be captured by CFD models [23]. Moreover, currently available CFD models still struggle to replicate large-scale bioreactor performance accurately and require substantial computational resources, resulting in very long real-time simulations, often taking weeks to months to simulate process snapshots lasting only hundreds of seconds [61]. Nevertheless, studies have shown CFD’s effectiveness in describing mixing in large-scale bioreactors, although verifying these models remains challenging due to experimental limitations [56]. To overcome the computational limitations, compartment models can be applied. Here, bioreactors are subdivided into interconnected, ideally mixed zones that approximate large-scale flow pattern, facilitating snapshot simulations within several seconds [61]. For a systematic evaluation of gradient estimation methodologies, including regime analysis, tracer studies, and CFD frameworks, the comprehensive review by Nadal-Rey et al. [61] provides critical insights into their application to industrial bioreactor hydrodynamics. To capture the dynamic conditions experienced from a cell’s perspective in industrial-scale bioprocesses, a so-called lifeline analysis can be performed [27, 28].

Given the high volume and possibly high levels of viscosity, the occurrence of gradients is to be expected in large-scale bioreactors. As a generalized rule regarding gradients of substrates consumed by cells in a bioreactor, they are likely to occur when the characteristic time of the substrate consumption or cellular reaction time (*τ*_*C*_) is equal to or lower than the characteristic time for transport or mixing time. For the estimation of the likelihood of substrate concentration gradients, *τ*_*C*_ is calculated by dividing the mean substrate concentration (*c*_*S*_) by the mean substrate consumption rate (${Q}_{S}={q}_{S}\bullet {c}_{X}$) [84]:

1 $${\tau }_{C}=\frac{{c}_{S}}{{q}_{S}\bullet {c}_{X}}$$

From Eq. (1), it is evident that higher biomass concentrations (*c*_*X*_) and biomass specific substrate consumption rates (*q*_*S*_) increase the potential for an abundance of gradients in large-scale bioreactors.

The mixing time in a bioreactor is defined as the duration required to homogenize the liquid in a bioreactor until it equally reaches, e.g., 95% (Φ_*95*_) of the final concentration after adding a tracer [86]. The importance of mixing time has been widely represented before [45, 46] as it is proportional to the tank diameter and can be a good choice to keep constant in scale-down bioreactors [25, 66]. Literature suggests that approximately 10% of large-scale bioreactors operated in aerobic bioprocesses face substrate-rich and oxygen-limited conditions, implying that cells may encounter these conditions for at least 10% of the circulation time, hence most likely causing byproduct formation [47].

*Substrate concentration gradients*. Substrate concentration gradients significantly negatively affect KPIs such as product yield and titer [27, 28, 90]. Local feeding of the limiting substrate into the bioreactor rises the likelihood of gradients. Feeding concentrated substrate may create spatial gradients, affecting the metabolic state of cells in the proximity of the point of feeding and consequently bioreactor efficiency. As substrate feeding typically occurs at a single point on top of the liquid phase a gradient is formed along the bioreactor’s height [81], with high substrate concentration at the top defined as excess zone, lower substrate concentration in the middle of the bioreactor, defined as limitation zone and low substrate concentration on the bottom defined as starvation zone [29]. In a study by Larsson et al. [48], *Saccharomyces cerevisiae* was cultivated in a 30 m^3^ STR with a working volume of 19.8–22.3 m^3^. The bioreactor was fed with a concentrated glucose solution of 600 g L^−1^. Significant substrate concentration gradients were observed with glucose concentration reaching 40.7 mg L^−1^ near the feed port at the top of the bioreactor and only 4.3 mg L^−1^ at the bottom. This represents a nearly tenfold higher substrate concentration at the feed port [48].

The cells in the bioreactor pass these zones stochastically with varying frequencies. Experiencing these fluctuating substrate concentrations impacts cell metabolism. Especially, the rapidly fluctuating high-concentration glucose gradients in the feeding zone cause the cells to consume glucose more quickly, therefore in addition initiating oxygen limitation in aerobic bioprocesses [22], and possibly overflow metabolism [81]. Thus, it is crucial to avoid substrate starvation, and excessive substrate concentrations in the bioreactor.

Strategies exist for mitigating substrate concentration gradients in large-scale bioreactors to enhance performance. These strategies include multi-point feeding and positioning the feeding port(s) close to the impeller region of a STR. Mixing is most efficient close to the impeller region due to the high local energy dissipation rate [2]. For instance, an in silico design optimization showed that placing the substrate feed location in an industrial scale STR closer to the impeller region can reduce penicillin yield loss from 18.4% to 8.6% with *Penicillium chrysogenum* [27, 28].

*Dissolved oxygen* (*DO*) *gradients*. In contrast to critical limits of substrate concentrations, the primary concern regarding DO is to maximize the oxygen transfer rate (OTR) to meet the culture’s oxygen demand [38]. This focus is justified since cells are unlikely to encounter DO levels high enough to cause physiologic effects due to the low solubility of oxygen in water, and the three-dimensional oxygen transfer from the homogeneously dispersed gas bubbles in the medium.

The dynamics of DO concentrations in bioreactors are critically influenced by the dispersion of the gas phase at the bottom of the vessel, and the effects of hydrostatic pressure. As gas bubbles ascend through the liquid medium, they become depleted in oxygen, leading to decreased DO concentrations at the gas–liquid interface [76]. Hydrostatic pressure intensifies this effect. The hydrostatic pressure decreases with the ascending gas bubbles, which reduces the partial pressure of oxygen even more. This results in a vertical gradient in DO at the gas–liquid interfaces reducing the local OTR approaching the liquid surface [87]. The height-dependent nature of OTR has important implications for bioprocess optimization. The issue of insufficient OTR is particularly prominent in viscous fermentation broths. Insufficient oxygen levels in the upper regions can adversely affect microbial activity and overall process efficiency [67].

With *E. coli* for instance, DO gradients have been reported to negatively affect the growth rate, and initiate general stress responses together with oxygen limitation responses at a single-cell level, such as induction of genes for anoxic growth [30]. The decreased growth rate might result from overflow metabolism products produced due to oxygen limitation [30].

*pH gradients*. Fewer studies have focused on extracellular pH gradients. Unlike gradients in substrates or DO, which tend to be more evenly distributed throughout the culture medium, pH gradients are generally more localized and short-lived, forming steep and transient hotspots near titrant addition points [44]. These hotspots are most pronounced near the addition ports of concentrated titration reagents for pH control, such as acids or bases [82]. Concentrated reagents are often used to minimize the liquid volume added to the bioreactor. However, the local pH in these regions can deviate strongly from the physiologic range of the cells, which can lead to metabolic stress and reduced bioprocess performance [2]. The buffering capacity of the culture medium helps to mitigate the extent and duration of these pH excursions, but cannot completely prevent them, especially with high titrant addition rates or suboptimal mixing conditions. Recent modeling and experimental work has shown that pH hotspots in large-scale fermentations can be up to one pH unit above or below the target value, depending on the bioreactor design and titrant strategy [53]. Deviations in pH have been reported to have a noticeable effect on the transcriptional response and enzyme activity of bacteria, leading to decreased biomass growth and product formation [55, 83]. For example, *Streptococcus thermophilus*, cultivated only 0.3 pH units away from its optimal pH, exhibited a 20% reduction in growth. This suggests that minor changes in pH can cause significant alterations in cellular metabolism [82]. In mammalian cell cultures, the pH of the medium is often controlled by CO₂ buffering, which helps stabilize the pH by maintaining the balance between CO₂ and bicarbonate ions. This buffering system is critical for controlling the pH within a range that supports optimal cell growth and productivity. However, because pH is closely coupled to CO₂ concentration, any significant deviation from the pH set-point can lead to corresponding changes in CO₂ levels, further complicating cellular metabolism [44]. Therefore, the importance of a precise pH control is significantly greater in mammalian cell cultures than in microbial cultures.

*CO*_*2*_*/HCO*_*3*_^−^ *gradients*. Dissolved CO_2_/HCO_3_^−^ gradients, influenced by hydrostatic pressure in large-scale bioreactors, can negatively impact cellular metabolism. Hydrostatic pressure increases with the scale of the vessel, resulting in higher concentrations of dissolved CO_2_/HCO_3_^−^ [45, 46]. The local concentrations of CO_2_ at the gas–liquid interface can be assumed to be in equilibrium, meaning that Henry’s law is applicable [58]. Part of the dissolved CO_2_ dissociates in water into bicarbonate (HCO_3_^−^) and H_3_O^+^, or into carbonate (CO_3_^2−^) and 2 H_3_O^+^ at higher pH values. Thus, the total solubility of CO_2_/HCO_3_^−^ becomes much higher in the fermentation medium at typical pH (pH 6.5–pH 7.5). CO_2_/HCO_3_^−^ levels can be especially high in cultures with elevated biomass concentrations or growth rates, as the cells generate substantial quantities of CO_2_ [57, 58]. In general, it is important to differentiate between elevated CO₂ levels and CO₂ gradients. Elevated absolute concentrations of CO₂ due to cell metabolism and pressure effects are known in large-scale processes. However, recent modeling studies [53], Sarkizi Shams [73] have shown that the spatial gradients of CO₂ within the bulk liquid are relatively small compared to other state variables such as DO or substrate concentration. Elevated levels of dissolved CO_2_/HCO_3_^−^ have been observed to act as metabolic inhibitors or transcriptional effectors, resulting in reduced cellular growth across various organisms, with pronounced effects on fungi and mammalian cells and more moderate influence on bacterial cultures (Blombach and Takors, [9]). It is assumed that growth reduction is attributed to osmolality shifts in the media (Blombach and Takors [9]). For example, at an industrially relevant residence time of 3.6 min under CO_2_/HCO_3_^−^ gradients, no considerable effect was observed on the process performance of *Corynebacterium glutamicum*, with growth and product formation being similar to control conditions. Nevertheless, transcriptional analysis revealed up to 66 genes that were differentially expressed within just 3.6 min under CO_2_/HCO_3_^−^ exposure [14]. These findings underline the potential physiologic sensitivity to transient CO₂ exposure, even if the absolute spatial gradient in large-scale reactors may be minor.

*Impact of gradients on different production hosts.* In summary, the impact of gradients in large-scale bioreactors is highly organism-dependent and should be carefully considered when designing scale-down studies. For bacteria as *E. coli*, rapid fluctuations in substrate and DO are particularly detrimental, as the cells react within seconds, often triggering overflow metabolism and stress responses. Consequently, *E. coli* requires scale-down configurations that mimic fast environmental transitions, such as STRs with dynamic feeding regimes or stirred tank bioreactor with plug-flow reactor (STR-PFR) combinations with short residence times. Other bacteria without showing overflow metabolism like *Corynebacterium glutamicum* display relatively stable productivity under short-term CO₂/HCO₃⁻ and DO fluctuations, although transcriptional responses indicate underlying physiologic changes. In contrast to bacteria with distinct overflow metabolism, mammalian cells like Chinese hamster ovary (CHO) cells are more sensitive to sustained variations in DO, pH, and CO₂/HCO₃⁻ levels due to their slower adaptation mechanisms. These gradients can lead to decreased viability, altered glycosylation patterns, and reduced product quality, necessitating scale-down approaches that replicate prolonged exposure, such as single multi-compartment bioreactors or coupled STR-STR systems. Yeast, e.g., *Saccharomyces cerevisiae* exhibit intermediate sensitivity, responding metabolically to both substrate and oxygen gradients. Interestingly, certain gradients may even enhance product yield under specific conditions, highlighting the nuanced interplay between environmental heterogeneity and cellular performance. Therefore, choosing an appropriate scale-down strategy depends not only on the type of gradient to be studied but also on the biologic response time and sensitivity of the production host. This will be further described in the following chapter.

## Scale-down bioreactor configurations

Scale-down bioreactors are a useful tool for investigating the effects of gradients in large-scale processes on a laboratory scale. However, researchers often face challenges in approaching full-scale conditions. A cell population cultivated in a scale-down bioreactor should display the same physiology and productivity as the bioprocess on a large-scale. Furthermore, a well-designed scale-down bioreactor should be user-friendly and have a low unit cost with high throughput while requiring minimal manual labor (Medeiros Garcia Alcântara and Sponchioni, [59]). It should also allow controlled generation of gradients that reflect the dynamics of large-scale processes [42, 67]. It is important to distinguish whether the entire population of cells is uniformly exposed to the simulated gradients or whether only subpopulations experience these environmental changes due to spatial or temporal distribution. There are distinct advantages and disadvantages to both synchronous and asynchronous gradient exposure. Uniform exposure simplifies the interpretation of cellular responses [62]. However, such uniformity does not accurately reflect large-scale bioreactors, where cells travel through spatially and temporally heterogeneous environments. In industrial bioprocesses, no single cell follows the same path or encounters identical conditions, making asynchronous exposure arguably more representative of real-world processes [27, 28, 87]. From this perspective, scale-down designs that allow only a subset of the population to be exposed to specific gradients at any given time may better replicate industrial performance, especially when population robustness or process stability is of interest [21, 30]. Therefore, the preference for synchronous or asynchronous exposure depends heavily on the study’s aim: mechanistic insight versus process mimicry.

In addition, the technical setup itself must be evaluated for potential artifacts. Components such as pumps, tubing, and valves can inadvertently affect cell physiology through mechanical stress or unintended flow patterns. The capability of scale-down bioreactors to replicate multiple gradients simultaneously, such as substrate concentration, DO levels, and pH fluctuations, which might potentially also happen indirectly, is another essential factor. Moreover, the extent to which the setup allows for the integration of real-time monitoring tools, such as online probes and sensors, determines the level of insight that can be achieved during experiments.

However, achieving the same physiology and productivity as the bioprocess on a large-scale requires many aspects to match, such as physical differences, fluid dynamics, and the bioprocess control strategy. Regardless of the challenges, scale-down bioreactors offer a convenient way to investigate the large-scale bioprocess gradients discussed above in a more defined environment, as executing such studies in large-scale bioreactors is often impractical, costly, and often not feasible due to limited availability [67]. Typically, scale-down studies are carried out by either employing combinations of bioreactors or with a single bioreactor and a special feeding regime [65]. These considerations provide a structured framework for the detailed analysis of various scale-down bioreactor configurations presented in this section.

**Fig. 2:**
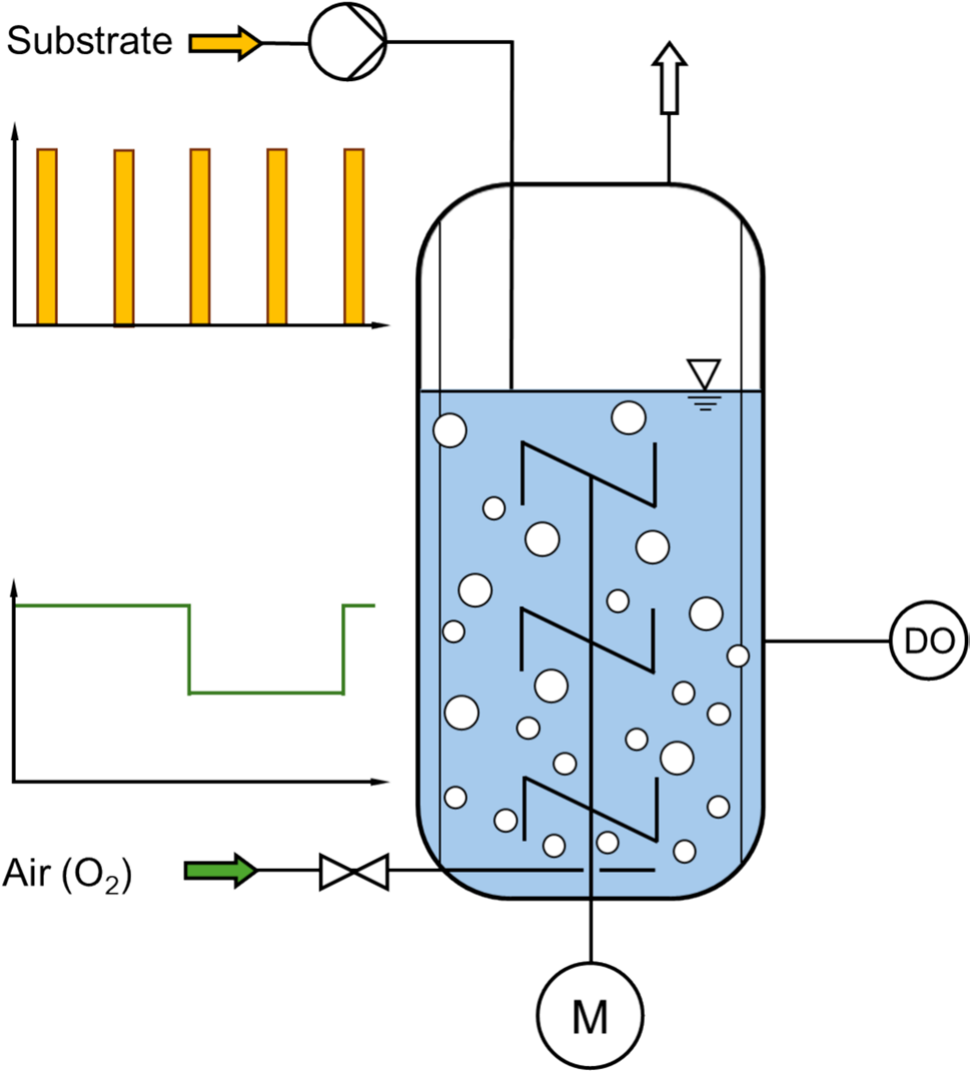
Scheme of a stirred-tank bioreactor operated as a fed-batch process with combined dynamic feeding regimes of substrate and oxygen supply used for scale-down studies

*Stirred-tank bioreactor with special feeding regimes*. Special feeding regimes in scale-down studies use dynamic strategies to analyze metabolic responses to gradients. This is a relatively straightforward way of scale-down studies, as a well-mixed STR is subjected to a special dynamic feeding regime creating concentration gradients in time instead of space, e.g., substrate concentration, DO level or pH (Fig. 2) [89].

In continuous cultivations, dynamic alterations in bioprocess conditions can be introduced either immediately after the start of the continuous phase, allowing investigation of whether and how a steady state is reached under fluctuating conditions, or after the establishment of a steady state, to study the systems response to controlled perturbations. These approaches are particularly useful for understanding microbial adaptation to environmental fluctuations, such as starvation and substrate excess. For example, Minden et al. [60] demonstrated how *S. cerevisiae* Ethanol Red™ gradually adapted to fluctuating glucose availability during limitation-starvation-limitation cycles, with gene-expression changes revealing nuanced physiologic responses beyond abrupt shifts.

In contrast, fed-batch bioreactors, mostly applied in industrial bioproduction, benefit from special feeding regimes to explore physiologic responses to intermittent substrate supply under more complex and non-steady-state conditions. In a fed-batch scale-down study by De Jonge et al. [19], *Penicillium chrysogenum* was subjected to intermittent substrate feeding. The results showed that penicillin production was reduced by a factor of two in the intermittently fed cultures compared to the constantly fed cultivations, while biomass yield stayed the same [19].

In another approach, the applicability of two *E. coli* triple reporter strain to detect phenotypic population heterogeneity was studied. These strains allow for the simultaneous monitoring of single-cell growth, general stress response, and oxygen limitation through the expression of fluorescent proteins. Both strains were cultivated in a STR, simulating industrial-scale conditions by varying growth rates and intermittent glucose feeding with turning off the aeration for 30 min. The results showed that both strains effectively reflected physiologic changes in response to environmental fluctuations highlighting the importance of understanding phenotypic population heterogeneity in bioprocesses [30].

Phenotypic population heterogeneity, defined as the variation in gene expression and metabolic states among genetically identical cells, has emerged as a central theme in bioprocess engineering. This phenomenon becomes particularly relevant in the presence of environmental gradients in industrial bioreactors, as well as under dynamic environmental changes encountered at lab-scale. While traditional scale-down studies often focus on average process parameters, it is increasingly recognized that subpopulations of cells may respond differently to environmental gradients, leading to complex process dynamics and potentially impacting product quality and yield. In this context, the use of fluorescent reporter strains offers valuable insights into how cells respond to fluctuating environments, factors that are often exaggerated in scale-down setups designed to mimic industrial-scale limitations [20, 30, 34, 35].

Bafna-Rührer et al. [5] studied the effect of glucose and oxygen feed oscillation in a miniaturized STR on *E. coli’*s transcriptional and physiologic responses by pulsed feeding of glucose and oxygen to mimic rapid fluctuations seen in industrial setups. This allowed the multiplexing of both nutrient and oxygen limitation in a controlled environment. Results showed that only glucose oscillation led to no significant decline in biomass formation but to an increase in carbon dioxide formation. In comparison, oxygen oscillating experiments led to a significant decrease in biomass formation by around 80% due to an accumulation of the by-product acetate. Combined oscillation of glucose and oxygen led to a decrease in biomass formation by around 50% compared to no oscillations and lower acetate accumulation compared to single oxygen oscillations. The researchers also observed that oxygen limitation caused persistent transcriptional changes, such as catabolite repression and activation of stress responses, while glucose starvation induced only transient stringent responses. Importantly, the interplay between oxygen and glucose oscillations produced unique disruptions in central metabolic pathways, highlighting complex, nonlinear interactions that cannot be captured by studying single-parameter fluctuations alone.

In a study by Serrato et al. [80], hybridoma cell cultures were cultivated in a 220 mL bioreactor and were exposed to DO concentration oscillating between 0 and 14% air saturation for various oscillating periods and compared with cell cultures where the DO concentration was kept constant at 10%. The results showed that the cells exposed to dynamic DO level changes exhibited decreased cell viability, increased by-product concentrations, and differences in protein glycosylation.

A similar study was conducted by Jiang et al. [37] to study the impact of pH excursion on Chinese hamster ovary (CHO) cells in a fed-batch operated STR. It was observed that the glycosylation pattern in antibodies produced by the CHO cells changed due to the frequent addition of base. From day 2 to day 8 of the cell culture, a total base volume equivalent to 2–6% of the cell culture was added. The researchers noted that pulse experiments in a single-compartment bioreactor could not accurately simulate the pH perturbation effect. This is because the pH pulses not only increased the pH of the entire bioreactor but also raised the osmolarity. As a result, it became challenging to determine the root cause of impaired cell growth [37].

Recent advances in single-cell microfluidic cultivation offer a complementary approach to conventional STR scale-down studies. Blöbaum et al. [8] presented a dynamic single-cell microfluidic cultivation (dMSCC) pipeline that allows quantification of microbial robustness and population heterogeneity under rapid feeding/starvation cycles (1.5–48 min) in *S. cerevisiae*. Their results showed that longer oscillation intervals decreased specific growth rates but increased ATP content, while rapid oscillations induced morphologic changes such as pseudohyphal growth. Importantly, the study demonstrated that population heterogeneity and functional stability can be systematically quantified at single-cell resolution, providing insights into cellular adaptation to dynamic environments similar to those in large-scale bioreactors.

**Fig. 3:**
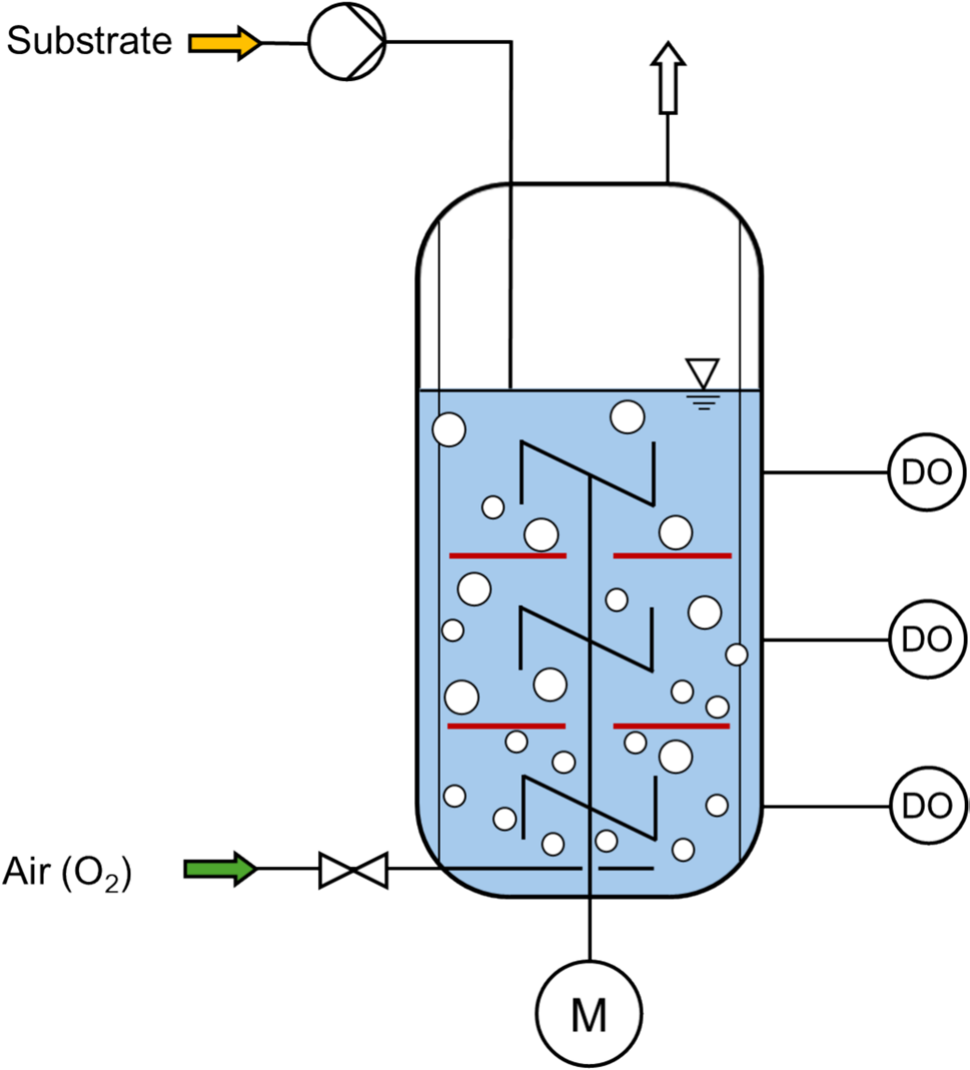
Scheme of a stirred-tank bioreactor operated as a fed-batch process with cylindrical disks (red) between the agitators (single multi-compartment bioreactor (SMCB) to enlarge mixing times. In the upper compartment, increased substrate concentrations occur at reduced DO concentration; in the lowest compartment, the situation is *vice-versa* (low substrate concentrations at high DO level)

*Stirred-tank bioreactor with internal compartments*. Stirred-tank bioreactors with internal compartments allow a controlled subdivision of the whole bioreactor volume into several compartments (Fig. 3). Usually, cylindrical disks with a small number of openings are installed in between the agitators along the rotating axes of the STR to increase the mixing time. Each compartment can be considered as well mixed, but the axial transport of the liquid phase with the dispersed gas bubbles is reduced.

In the study by Schilling et al. [75] a single-multicompartment bioreactor (SMCB), consisting of a STR with internal compartments, was designed to explore the effects of mixing time on fermentation processes, using *Corynebacterium glutamicum DSM 5715*, a leucine-auxotrophic strain, for the aerobic production of L-lysine. A 42-L STR was equipped with six Rushton turbines separated by five cylindrical disks to mimic large-scale fermentation conditions. This setup resulted in a mixing time that was 13 times longer than that in a standard 42-L STR. The cells cultivated in the bioreactor with internal compartments demonstrated a reduction in sugar consumption by 14%, ammonium consumption by 19%, and biomass formation by 7%. Furthermore, L-lysine formation decreased by 12%.

In another more recent example, a SMCB was introduced to study scale-up behavior of mammalian cell culture processes. The SMCB was used to mimic characteristics as long mixing times and DO gradients observed in industrial scale bioreactors by adjusting volumetric power inputs ranging from 1.5 to 20.4 W m^−3^. The SMCB was equipped with a stirrer combination of a Rushton turbine at the bottom and a pitched-blade turbine separated by a compartment disk to create two similar-sized compartments. The results from fed-batch cultivations of CHO cells revealed that the SMCB conditions led to an increase in lactate accumulation by 87%, higher glucose uptake, and a reduction in viable cell concentrations during the stationary phase, all typical of large-scale processes. This study highlights the potential of a SMCB as a powerful tool for predicting scale-up challenges and understanding specific drawbacks in cell line performance at the early stages of process development [24].

**Fig. 4:**
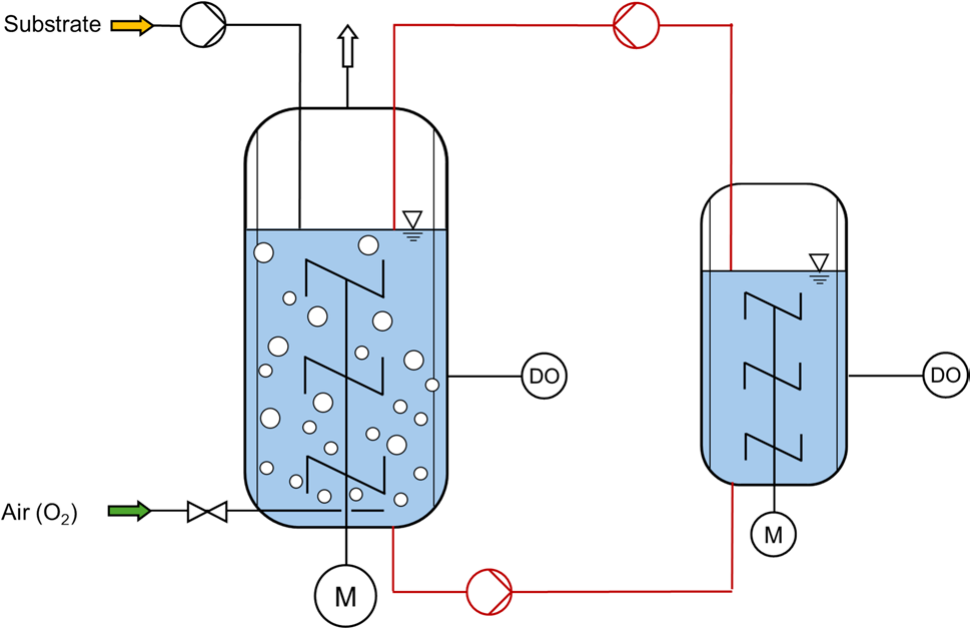
Scale-down bioreactor configuration with two stirred-tank bioreactors operated as a fed-batch process. Here, a smaller STR is operated in a bypass (red) without gassing to simulate short-term oxygen depletion. At low substrate concentrations in the scale-down bioreactor setup, an additional substrate limitation may occur

*Combination of multiple stirred-tank bioreactors*. Scale-down bioreactor configurations with two STRs coupled to each other are frequently applied (Fig. 4). Usually, the cell suspension is recycled in between two well-mixed STRs, operated at varying reaction conditions, e.g., pH gradients, DO gradients.

To study the effect of substrate fluctuations on a fed-batch process with baker’s yeast as an example, the glucose feed was solely added in one of the STRs, leading to a higher glucose concentration than in the second compartment. Different studies were conducted in the two-fermenter system with varying circulation rate and volume ratios between both STRs. The authors observed a reduction in biomass yield and an increase in ethanol concentration, acetic acid, and glycerol formation due to imperfect mixing compared to the cultivation in a single STR [85].

Sandoval‐Basurto et al. [72] conducted a study using a two-compartment scale-down system, consisting of two interconnected 1.5 L STRs. One bioreactor was kept in anaerobic conditions, while the other maintained a DO of at least 6% air saturation. *E. coli* cells were continuously circulated between the vessels, simulating circulation times ranging from 20 to 180 s. The researchers observed that even brief anaerobic exposure significantly impaired cell growth and recombinant pre-proinsulin production. The specific growth rate of the cells decreased with increasing circulation times, with a maximum reduction of 30% at the highest circulation time tested. These findings highlight the negative effects of transient anaerobic conditions, likely in large-scale operations, on the performance of *E. coli* in recombinant protein production.

The study of Brunner et al. [13] investigates the influence of pH inhomogeneities on CHO cell physiology and performance in a fed-batch process using a STR-STR scale-down reactor consisting of a 3 L STR and a 0.7 L STR. This approach should mimic pH gradients by exposing cells alternately to zones with controlled pH perturbations (overshoot to pH 9) and a well-mixed zone at pH 7.2. The results demonstrated that extracellular pH changes immediately lead to intracellular pH increases, possibly driven by CO_2_ stripping from the media, which in turn alkalized the cells. Key findings were that pH fluctuations reduced specific growth rates, leading to lower maximum viable cell densities and decreased product titers compared to single-compartment controls.

Moreover, configurations comprising three subsequent STRs can be implemented, allowing multiple scenarios with diverse conditions to be tested simultaneously, e.g., three industrially relevant metabolic regimes regarding substrate availability: excess, limitation and starvation zones. Thereby, each of the three bioreactors represented one of these regimes [98].

In a study by Buchholz et al. [14] a 100 L STR followed by two 1 L-scale STR were arranged in a loop. The pCO_2_ in the main bioreactor was set to 50 mbar, whereas in the cascade bioreactors the pCO_2_ was set to 150 mbar, and 315 mbar, respectively. These gradients should simulate the conditions in industrial scale bioreactors with poor mixing. It was found that fluctuating CO_2_ and bicarbonate levels triggered rapid and significant transcriptional responses in *C. glutamicum*. These responses highlighted the microorganism’s ability to adjust its gene expression to cope with simulated large-scale CO_2_ gradients.

However, there are also several studies showing increased bioprocess performance in response to glucose fluctuation. Wright et al. [96] studied the continuous production of insulin using *Saccharomyces cerevisiae* with two interconnected 0.5 L STRs. The working volumes of both bioreactors were kept constant at 170 mL, and 350 mL, respectively, during the continuous phase of cultivation with a dilution rate of 0.1 h^−1^. The cascade of the two STRs simulated two zones of industrial-scale bioreactors: one near the substrate inlet, which has a high substrate concentration and lower oxygen concentration, and another substrate-limited zone, with a high oxygen concentration. The gradients with short mixing times of 0.4 min in the two-compartment bioreactor system resulted in a minor reduction in biomass yield but an increase in product yield during the first 150 h of continuous cultivation reaching a maximum of around 1.60 a.u. compared to only approximately 1.10 a.u. after 50 h of continuous cultivation. After the maximum product yield was reached, a reduction of protein yield was observed [96]. Hence concluding that the gradients can be sometimes beneficial for productivity.

**Fig. 5:**
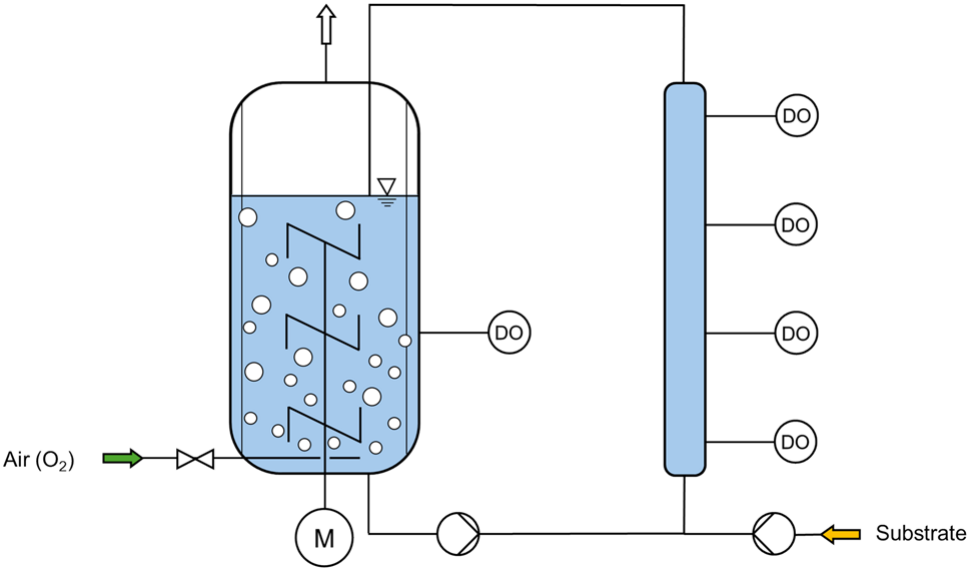
Scale-down bioreactor configuration combining a stirred-tank bioreactor with a plug flow reactor (PFR) operated as a fed-batch process. Here, the PFR is operated in a bypass with substrate supply to simulate short-term substrate excess with DO gradients. Sampling along the PFR, indicated by probes for the measurement of DO as an example enables the measurement of gradients and short-term physiologic reactions of the cells

*Combination of a stirred-tank bioreactor with plug flow bioreactor(s).* Combining a STR with one or several plug flow reactors (PFR) in a bypass allows for the study of short-term substrate concentration gradients and their effects on process performance. This configuration, often used in scale-down studies, helps simulate the dynamic nutrient conditions encountered in large-scale bioprocesses (Fig. 5) [19, 49]. These gradients can induce phenotypic population heterogeneity, where genetically identical cells exhibit diverse responses, such as varying growth rates and productivity. Understanding this variability is crucial for optimizing bioprocess efficiency and product consistency [35].

However, a critical limitation remains in achieving a narrow residence time distribution in the tubular bypass at laboratory scale, as this criterion ensures that all fluid elements spend the same amount of time in the plug flow reactor (PFR) [10]. In a straight tubular bioreactor a narrow hydraulic residence time distribution is only achievable in turbulent flow, where radial mixing is sufficient to minimize hydraulic residence time disparities [6]. Achieving turbulent flow in a straight tubular bioreactor at lab scale would lead to short hydraulic residence times, often lower than typical circulation times in large-scale bioreactors, which restricts accurate down-scaling of industrial bioreactor conditions [3, 33, 42, 95]. Typical circulation times on a multi-minutes scale can then only be achieved in the tubular bypass with laminar flow, characterized by the typical parabolic flow velocity profile as function of the radius, where fluid elements closer to the center move much faster than those near the walls, resulting in a very broad hydraulic residence time distribution [70].

**Fig. 6:**
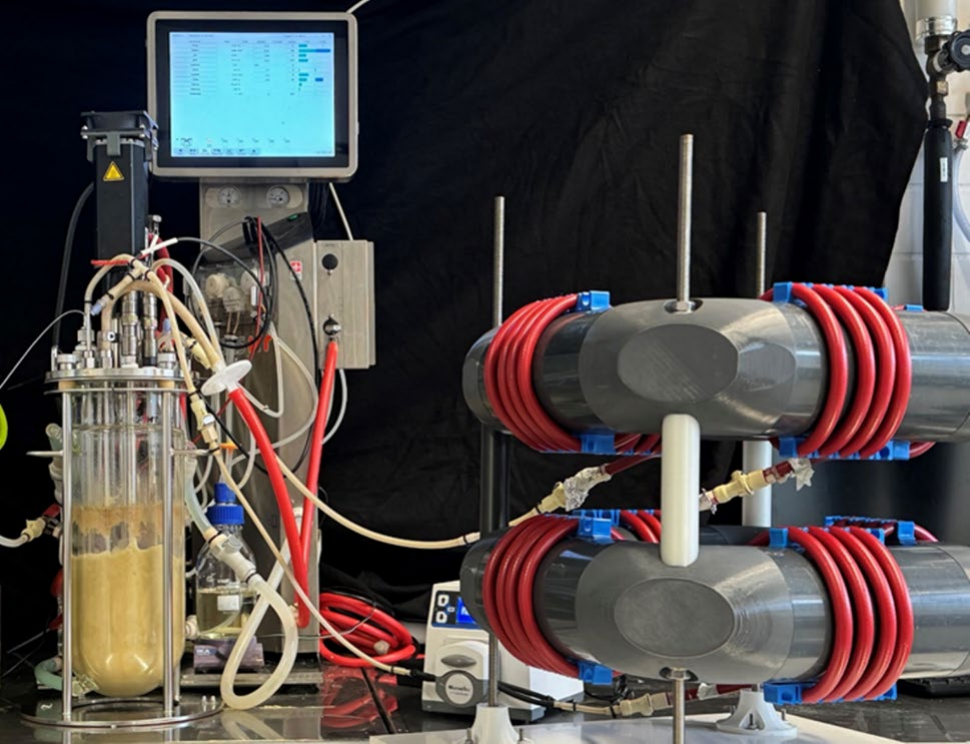
Photograph of a coiled flow inverter (on the right with red tubing) operated as a tubular bypass (with a total volume of 0.4 L) to a 3.6 L-scale stirred tank bioreactor enabling mean residence times of 1–10 min with narrow residence time distributions (Bodenstein number Bo >  > 100). (Photo provided by Prasika Arulrajah, Technical University of Munich)

A solution is the application of a coiled flow inverter, which can be operated under laminar flow conditions as a tubular bypass (Fig. 6). Coiled flow inverters use coiled tubing segments interspersed with 90° bends to generate and reorientate Dean vortices (paired vortex structures produced by centrifugal forces as the fluid navigates coiled sections) in the laminar flow regime. The periodic reorientation through 90° bends promotes narrow hydraulic residence time distributions. This has been successfully proven recently by applying a coiled flow inverter in a bypass to a STR at a mean hydraulic residence time of 102 s [35].

To investigate the effect of substrate-rich conditions and oxygen limitation separately, Lara et al. [47] employed a 3 L STR combined with a PFR of 3.5-mL volume. The glucose feed was mixed with the broth coming from the STR and fed to the PFR to simulate glucose-rich environments in large-scale bioreactors. The PFR was equipped with 11 ports for controlled sampling at different distances from the inlet. Samples were taken over 92 s of the mean residence time in the PFR, with the first five samples taken within the first 20 s to ensure aerobic conditions. Also, the PFR had two channels: one for media circulation and another for air circulation, separated by a semipermeable membrane that allowed O_2_ and N_2_ transfer, thus inducing aerobic or anaerobic conditions. During the first 10 s in the PFR, acetate, and formate were produced at a higher rate than during the remaining 82 s in the PFR. Acetate production in *E. coli* is mainly induced by glucose overflow. In glucose-rich anaerobic conditions, the glucose uptake rate doubled, resulting in the accumulation of organic acids such as acetate, formate, ethanol, lactate, and succinate.

Neubauer et al. [64] studied the dynamics of a *E. coli* K-12 strain W3110’s response to oscillatory changes in the glucose concentration during fed-batch cultivation. For that, a two-compartment bioreactor, consisting of an STR and an aerated PFR with a mean residence time of 113 s was used. When repeated short-term glucose starvation was introduced, guanosine-3'-diphosphate5’-diphosphate, a glucose starvation alarmone (ppGpp), was produced by *E. coli*. This alarmone causes drastic reduction of all cellular reactions for cell growth and reproduction and prepares the cell for survival by concentrating the reserves [64].

Anane et al. [4] studied systematically the effect of DO gradients and mixing times on a CHO cell line expressing a monoclonal antibody during a fed-batch process in a scale-down bioreactor. The scale-down bioreactor consisted of a 5 L STR and a 1.07 L PFR connected with a centrifugal pump with magnetically levitated contactless impeller, where mean residence times within the PFR ranging from 70 to 120 s were studied. For a mean residence time below 90 s in the PFR, in response to DO gradients, the cells produced lactate at steady rate up to day 7, and the lactate got reassimilated after day 7. They also observed, that the viable cell density declined significantly during the stationary phase in the scale-down bioreactor, with up to 30% reduction at mean residence times ≥ 90 s in the PFR compared to the cultivation in a well-mixed STR. In addition, host cell protein impurities increased with prolonged exposure to DO gradients, indicating heightened cell fragility and lysis, which may complicate downstream processing [4].

In a study by George et al. [26] a scale-down bioreactor consisting of a 15 L STR and a 0.85 L PFR in a bypass was applied to simulate the sugar (glucose and fructose) concentration gradients observed in industrial-scale baker’s yeast production. This setup allowed them to intermittently expose about 10% of the yeast culture to high sugar concentrations (0.45–1.9 g L^−1^) for 60-s periods in the PFR, mimicking the heterogeneous conditions found in large-scale bioreactors. The scale-down experiments revealed several key findings, including a 6–7% reduction in biomass yield, which was also observed in the large-scale 215 m^3^ bubble column bioreactor, compared to the homogeneous STR.

Olughu et al. [69] studied the effect of gradients (DO, pH, and glucose) in a two-compartment scale-down bioreactor (STR-PFR) using a recombinant *Corynebacterium glutamicum* strain producing L-cadaverine, a platform chemical used for the production of biopolymers. They studied the effect of intensifying gradients by incorporating both the residence time of cells in the PFR and the mean frequency at which the bacterial cells entered the PFR. They observed that the cadaverine production decreased by 59%, and the carbon dioxide production increased by 3.1-fold with a mean residence time of 5 min in the bypass [69].

In another study by Junne et al. [39], *Bacillus subtilis* was cultivated in a fed-batch process using a two-compartment bioreactor consisting of a 10 L STR connected to a 1.8 L PFR with four static mixer modules. The PFR is furthermore insulated and equipped with five sample ports, pH, and DO sensors along its length, allowing for analysis of gradients. Key findings include that oscillating substrate and DO concentrations led to a reduced glucose uptake rake by 10% and a sixfold increase in overflow metabolite accumulation (reduced alcohols and ethanol) compared to non-oscillating conditions.

In a STR, there is a sudden change in the environment when the cells enter the bioreactor, whereas in a plug flow bioreactor (PFR), gradual DO, pCO_2,_ pH or substrate gradients can be introduced. The study of Limberg et al. [52] compares two scale-down bioreactor setups: a two-compartment system with two STRs and a system combining a STR with a PFR. The first setup, based on a parallel fermentation system, consisted of two interconnected STRs with a combined working volume of 1 L. Thereby the volumetric distribution was 78% in the first STR and 22% in the smaller STR. For the STR-PFR configuration the total volume was 5 L. Here, a STR was connected with a custom-built PFR made of a thermally insulated stainless steel pipe with 18% of the total volume. They observed that both the STRs and STR-PFR combination led to similar l-lysine yields with *C. glutamicum*, even though they presented different flow regimes. Interestingly, hydrodynamic differences led to different byproduct formation levels. They found that at a wide range of mean residence times in the bypass STR, the most abundant byproduct was lactate, whereas in the combination of STR and PFR, more l-glutamate was produced as byproduct. They suggested that oxygen deprivation is weaker in a combination of STR bioreactors because, due to back mixing, some oxygen is always transferred from one STR to another [52].

In three-compartment scale-down reactor systems, such as the PFR-STR-PFR, it is possible to observe the effect of two different zones simultaneously. Lemoine et al. [49] designed a PFR-STR-PFR to study the impact of both substrate and oxygen gradients with *Corynebacterium glutamicum.* In this scale-down bioreactor configuration, there is the aerated STR and a PFR 1. The feed is injected at the bottom of the PFR 1, simulating an oxygen gradient. The STR is also connected to another non-aerated PFR 2, which is not provided with substrate, simulating dual substrate and oxygen limitation. It was observed that compared to a two-compartment scale-down configuration (STR–PFR 1), a three-compartment scale-down system resulted in the production of different metabolic byproducts produced by *C. glutamicum*. The concentration of byproducts such as succinate and lactate and certain amino acids was high in the three-compartment setup in comparison to two-compartment configuration due to prolonged exposure to oxygen limitation and increased energy charge value [49].

Marbà-Ardébol et al. [54] also did a similar experiment inducing substrate and oxygen gradients in a three-compartment bioreactor setup (PFR-STR-PFR) using *Saccharomyces cerevisiae.* It was observed that prolonged exposure of cells to oxygen limitation caused acetate accumulation and limited the conversion of sterol intermediates to final sterol products [1], Marbà‐Ardébol et al. [54]).

Looking forward, a stronger alignment with industrial practices is crucial. Access to real process data would significantly enhance model validation and reliability. For example, collaboration with industry partners could bridge the gap between laboratory-scale findings and industrial applications, ultimately accelerating the optimization of large-scale bioprocesses.

Key findings of the scale-down bioreactor studies described before are summarized in Table 1.

**Table 1:** Comparative summary of scale-down bioreactor experiments: setup, organisms, and key observations (Bo Bodenstein number)

References	Scale-down configuration	Organism	Main gradient simulated	Key findings
[5]	STR with pulsed feeding and aeration	*E. coli*	Glucose and DO	Combined oscillations reduced biomass by ~ 50%; oxygen-only reduced it by 80%
[30]	STR + intermittent feeding + aeration off	*E. coli *(reporter strain)	Glucose and DO	Detected single-cell level heterogeneity reflecting physiologic changes in response to environmental fluctuations
[24]	Single multi-compartment STR	CHO cells	DO and mixing time	Increased lactate accumulation (87%), lower viability
[96]	STR–STR cascade	*S. cerevisiae*	Substrate and DO	~ 45% increase in insulin yield after 150 h of exposure to fluctuating gradients
[4]	STR–PFR	CHO cells	DO	Residence times ≥ 90 s led to increased lysis and host cell protein impurities
[35]	STR + CFI	*E. coli* (reporter strain)	Mixing and substrate	Achieved narrow residence time distribution (Bo > > 100); improved gradient simulation
[69]	STR–PFR	*C. glutamicum*	Glucose, DO, pH	59% lower product titer and 3 × higher CO₂ output under intensified gradients
[52]	STR–STR vs. STR–PFR	*C. glutamicum*	Glucose and DO	STR–PFR produced more L-glutamate; STR–STR resulted in more lactate formation

## Comparison of scale-down bioreactor configurations

The advantages and disadvantages of various scale-down bioreactor concepts depicted above (Table 1) will be summed up and discussed in the following.

*Single bioreactor configurations for scale-down studies*. Both, a STR with special feeding regimes, or a STR with internal compartments represent single bioreactor configurations. They can be operated as fed-batch or in continuous mode. The main advantage of a single bioreactor configuration is its simplified design and operation, making it easier to construct, maintain, and use compared to more technically complex combination of bioreactors. In addition, what differentiates this setup from the others is that all cells within the bioreactor experience the same gradients within a defined observation time, whereas, in other multi-bioreactor setups, only a portion of the cells is exposed to different environments. Furthermore, the cells within single bioreactor configurations are not exposed to additional shear stress or less defined reaction conditions caused by pumps and tubings needed with multi-bioreactor combinations.

The main advantage of a single bioreactor configuration for scale-down studies with all cells exposed to the same gradients may become a major drawback if combinations of varying reaction conditions need to be tested on a laboratory scale to mimic varying zones inside industrial scale bioreactors. Examples are substrate starvation, substrate limitation, and substrate excess combined solely in one of these zones with high or low oxygen supply or with high or low pH due to single-point addition of the titration agent (base or acid). In this case, multi-bioreactor setups are necessary, like combinations of STRs or STR and PFR(s).

*STR-STR scale-down bioreactors*: One of the major advantages of STR-STR systems is the independent control of different reaction conditions and combinations thereof in each compartment, enabling more realistic physical simulation of industrial-scale gradients [52]. Another advantage is that the residence time distributions in lab-scale STRs are well defined. Also, in STR-STR scale-down systems, various probes can be easily installed, allowing for convenient monitoring and sampling. Furthermore, it is easy to maintain different working volume ratios between the compartments [1].

One disadvantage of STR-STR scale-down setups is the limited spatial gradient representation. With this scale-down setup, only two zones can be simulated, exposing the cells to varying reaction conditions with a step-function when cells are exchanged between the STRs. This may oversimplify the gradients existing in industrial-scale bioreactors [68].

The combination of two STRs also results in a simplified lifeline of cells, which means that cells are not going through “excess”, “limitation”, and “starvation” zones, which are typical in industrial-scale bioreactors, but in this case only from “excess” to “limitation” [29]. More complex scale-down bioreactor configurations like STR-STR-STR combinations are necessary to depict these three zones.

The most challenging issue with STR combinations as scale-down bioreactors is the high level of back mixing, providing a very broad range of residence times of the cells in each compartment [52].

*STR-PFR scale-down bioreactors*: The STR-PFR are among the most commonly employed configurations. Due to the low back mixing, if operated correctly, the PFR ensures a narrow residence time distribution, exposing all cells passing this compartment for nearly the same time to the same reaction conditions. In addition, sampling ports at defined positions along the PFR allow for accurate space–time resolution, enabling detailed monitoring of process dynamics at various positions within the PFR, if back-mixing is low [40, 52]. Space–time is defined as the ratio of the bioreactor volume to the volumetric flow rate, representing the average time a fluid element spends in the bioreactor [50]. Importantly, space–time sampling in the PFR provides a powerful tool for dissecting dynamic bioprocess phenomena. Since each sampling port along the PFR corresponds to a specific space–time, researchers can map cellular responses to transient environmental gradients, such as substrate depletion or DO levels as a function of elapsed time in the PFR [99]. Furthermore, a PFR has a gradient along its length, allowing for observing metabolic answers to gradual changes in substrate concentrations, DO levels or pH value. An STR-PFR scale-down bioreactor can successfully simulate local heterogeneities existing in large-scale bioreactors, such as highly concentrated single-point substrate feeding [64].

However, potential limitations arise when dealing with longer mean residence times in the PFR, as extending the length of a PFR in the turbulent flow regime is not a feasible solution. A coiled flow inverter, which can be operated under laminar flow conditions as tubular a bypass is an approach to compensate for these challenges [35].

A major drawback of STR-PFR combinations is the restricted control of several gradients independently in the PFR. Feeding the concentrated substrate into the PFR will result in oxygen limitation and pH changes within a few seconds at the typical biomass concentrations of industrial production processes. Direct air gassing of the tubular bypass to overcome oxygen limitation may result in not well-defined residence time distributions of the cells due to the heterogenic bubble flow, or even worse, a slug flow of the gas phase, due to the typically small diameter of the lab-scale PFR.

Advantages and disadvantages of scale-down bioreactor setups are compared in Table 2.

**Table 2:** Comparative analysis of different scale-down bioreactor setups highlighting their advantages and disadvantages

	Description	Advantages	Disadvantages
Single STR	STR with controlled dynamic feeding (pulses) to create temporal concentration changes (oscillations)	Simplified design; cost-effective;	Limited spatial gradients may oversimplify real large-scale conditions
STR with internal compartments	STR divided into multiple compartments with disks to simulate poor mixing conditions and create spatial gradients	Controlled spatial gradients; enables increased mixing times	Complex design; requires careful configuration for accurate gradient simulation
STR-STR	Two STRs in loop, each simulating different zones (e.g., high substrate concentration in one, limitation/starvation in the other)	Independent control of varying reaction conditions and combinations thereof in each compartment; easy sampling	Typically two zones but more possible with added complexity; high level of back-mixing results in a broad range of residence times of the cells in each compartment; requires pump for broth transfer, introducing shear stress; requires frequent liquid additions for control (e.g., pH)
STR-PFR	Combines an STR with a plug flow reactor (PFR) to simulate gradients along the length of the PFR	Narrow residence time distribution in the PFR; supports detailed space–time resolution sampling	Restricted independent control of several gradients in the PFR; requires pump for broth transfer, introducing shear stress; requires frequent liquid additions for control (e.g., pH)

## Outlook: miniaturized bioreactors for scale-down studies?

Miniaturized and automated bioreactors have emerged as valuable tools in bioprocess development, offering a cost-effective and efficient platform for high-throughput experimentation, essential for identifying optimal bioprocess conditions or screening for new production strains (e.g., Hemmerich et al. [31], Hortsch et al. [36]; Tajsoleiman et al. [86]). Miniaturized bioreactors are often coupled with a pipetting robot (liquid handling station) for parallel operation [7]. The miniaturization, parallelization, automation, and digitalization of bioprocesses (e.g., [11], von den [92]) enables efficient early-stage bioprocess development, allowing researchers to rapidly evaluate bioprocesses by simultaneous execution of multiple experimental conditions [91]. Scalability of miniaturized bioreactors is the key issue in bioprocess development, exemplarily shown for the scale-up of high-cell density yeast fermentations from automated mL-scale STRs to the L- and m^3^-scale [77]. The high number of fully controlled and parallel-operated miniaturized bioreactors may enable their application as multi-STR cascades simulating gradients for scale-down studies in the future. Continuous operation of STRs on a mL scale has already been shown [78].

Recent studies have furthermore shown that miniaturized bioreactors can effectively replicate dynamic and heterogeneous environments existing in large-scale bioreactors. For instance, Anane et al. [3] demonstrated that parallel mini-bioreactors can replicate substrate gradients and allow dynamic perturbation studies. These configurations enable investigations of metabolic adaptations and regulatory responses under conditions that mimic large-scale bioreactor environments, supporting their use in scale-down studies.

Recent developments in microfluidic systems have further expanded the potential of miniaturized bioreactors for scale-down studies. A major advantage of microfluidic systems is their ability to precisely manipulate environmental conditions, which are fully decoupled from cellular activity, to study physiologic reactions and gene expression under fluctuating environments [88]. Recent works have explored the use of dynamic microfluidic single-cell cultivation as a novel scale-down approach. Täuber et al. [88] were able to investigate the effect of pH oscillation on microbial growth, providing insights comparable to the more common multi-compartment bioreactor experiments. Despite these advantages, microfluidic systems also face inherent limitations. Due to the extremely small working volumes and low number of cells, the range of applicable analytical methods is restricted. While microscopy is well-suited for single-cell studies in these systems, more comprehensive analyses are generally constrained [43].

Emerging technologies are starting to bridge the gap between laboratory and industrial-scale bioprocesses. One particularly promising example is a down-scale system (pDS, P4B GmbH, Wolkersdorf, Austria), which uses a combination of generative AI, digital twins, and 3D printing to design lab-scale bioreactors that try to mimic the hydrodynamic and gradient conditions of large-scale vessels. Unlike traditional designs that must compromise between key state variables (e.g., mixing time, mean power input, or tip speed), these AI-generated geometries allow for the decoupling of these constraints. As a result, lab-scale bioreactors can be custom-built to approximate the environment of, for instance, a 10,000 L STR. Looking forward, such specifically designed bioreactors show promise as tools for derisking scale-up, and potentially reducing the need for costly pilot-scale experiments. With further development, this approach could contribute to reshaping early-stage bioprocess development by integrating predictive physical modeling into routine lab workflows [74]. 

## Conclusion

Key operational challenges in large-scale bioreactors, such as gradients due to insufficient mixing, and reduced productivity, can be addressed with scale-down studies. Scale-down bioreactors are crucial for replicating large-scale experimental challenges on a smaller scale, allowing for detailed investigation and optimization of the process. In addition, scale-down bioreactors provide valuable insights into metabolic adaptability, stress response, and physiology. Scale-down bioreactors are thus particularly useful for studying microbial robustness under conditions like overflow and nutrient starvation, which ultimately affects the microorganism’s suitability for industrial application. 

Different scale-down setups can be utilized depending on the specific problem being addressed. There is no optimum scale-down bioreactor configuration. Each individual setup introduces both advantages and challenges, along with different producer cells and gradients to be studied. Scale-down approaches, taking into account CFD modeling and lifeline analysis of large-scale bioprocesses, remain essential for refining process conditions and ensuring that scale-down bioreactor configurations maintain the most important characteristics of their larger counterparts.

## Data Availability

No datasets were generated or analysed during the current study.
